# Interleukin-1 and the NLRP3 inflammasome in COVID-19: Pathogenetic and therapeutic implications

**DOI:** 10.1016/j.ebiom.2022.104299

**Published:** 2022-10-06

**Authors:** Nicola Potere, Marco Giuseppe Del Buono, Roberto Caricchio, Paul C. Cremer, Alessandra Vecchié, Ettore Porreca, Daniela Dalla Gasperina, Francesco Dentali, Antonio Abbate, Aldo Bonaventura

**Affiliations:** a Department of Medicine and Ageing Sciences and Department of Innovative Technologies in Medicine and Dentistry, G. D'Annunzio University, Chieti, Italy; b Department of Cardiovascular and Thoracic Sciences, Fondazione Policlinico Universitario A. Gemelli IRCCS, Catholic University of the Sacred Heart, Rome, Italy; c UMass Chan Medical School, Worcester, MA, USA; d Department of Cardiovascular Medicine, Heart, Vascular, and Thoracic Institute, Cleveland Clinic, Cleveland, OH, USA; e Medicina Generale 1, Medical Center, Ospedale di Circolo e Fondazione Macchi, Department of Internal Medicine, ASST Sette Laghi, Varese, Italy; f Department of Medicine and Surgery, University of Insubria, Varese, Italy; g Robert M. Berne Cardiovascular Research Center and Division of Cardiovascular Medicine, Department of Medicine, University of Virginia, Charlottesville, VA, USA

**Keywords:** SARS-CoV-2, COVID-19, NLRP3 inflammasome, IL-1β, IL-18, C-reactive protein, Anakinra, Canakinumab, Colchicine

## Abstract

A hyperinflammatory response during severe acute respiratory syndrome coronavirus 2 (SARS-CoV-2) infection crucially worsens clinical evolution of coronavirus disease 2019 (COVID-19). The interaction between SARS-CoV-2 and angiotensin-converting enzyme 2 (ACE2) triggers the activation of the NACHT, leucine-rich repeat, and pyrin domain-containing protein 3 (NLRP3) inflammasome. Enhanced inflammasome activity has been associated with increased disease severity and poor prognosis. Evidence suggests that inflammasome activation and interleukin-1β (IL-1β) release aggravate pulmonary injury and induce hypercoagulability, favoring progression to respiratory failure and widespread thrombosis eventually leading to multiorgan failure and death. Observational studies with the IL-1 blockers anakinra and canakinumab provided promising results. In the SAVE-MORE trial, early treatment with anakinra significantly shortened hospital stay and improved survival in patients with moderate-to-severe COVID-19. In this review, we summarize current evidence supporting the pathogenetic role of the NLRP3 inflammasome and IL-1β in COVID-19, and discuss clinical trials testing IL-1 inhibition in COVID-19.

## Introduction

Even though most subjects with coronavirus disease 2019 (COVID-19) experience an asymptomatic or pauci-asymptomatic clinical course, up to 10–15% of patients develops respiratory failure requiring hospitalization. Approximately 5% progresses to acute respiratory distress syndrome (ARDS), many with widespread thrombosis (arterial, venous, microvascular) and multiorgan failure.^1^^,^^2^ A dysregulated innate immune response to the virus, characterized by increased levels of pro-inflammatory cytokines contributing to pulmonary damage and hypercoagulability, is the hallmark of severe-to-critical COVID-19.^3^, ^4^, ^5^, ^6^

The NACHT, LRR, and PYD domains-containing protein 3 (NLRP3) inflammasome is a macromolecular platform that senses tissue injury and, in response, processes active interleukin-1β (IL-1β) and IL-18, two major pro-inflammatory cytokines, and can induce an inflammatory form of cell death termed pyroptosis. While an adequate innate immune response limits pathogen dissemination, exaggerated NLRP3 inflammasome activation drives hyperinflammation and triggers the coagulation cascade, thereby aggravating tissue injury.^7^ Viruses including the betacoronaviruses severe acute respiratory syndrome coronavirus (SARS-CoV) and Middle East respiratory syndrome coronavirus (MERS-CoV) are known to activate the NLRP3 inflammasome in host cells.^8^, ^9^, ^10^, ^11^ Early in the COVID-19 pandemic, the NLRP3 inflammasome and IL-1β garnered considerable attention due to their well-known biological functions in health and disease.^12^ Mechanistic studies have demonstrated that severe acute respiratory syndrome coronavirus 2 (SARS-CoV-2) potently triggers NLRP3 inflammasome assembly.^12^^,^^13^ IL-1β and IL-18 are significantly elevated in patients with severe COVID-19, and positively correlate with adverse clinical outcomes.^13^, ^14^, ^15^ Accordingly, post-mortem analysis of lungs from patients with COVID-19-related ARDS has shown intense expression of active NLRP3 inflammasome.^16^ Furthermore, common comorbidities (e.g., obesity, diabetes mellitus, heart failure [HF], hypertension) associated with poor COVID-19 evolution are characterized by basal inflammasome overactivation and persisting inflammation.^17^ These findings point out the existence of a vicious spiral starting with SARS-CoV-2 infection and unopposed NLRP3 inflammasome activation with subsequent excessive IL-1β production, leading to poor clinical outcomes. Based on these observations, it has been hypothesized that therapeutic strategies targeting the NLRP3 inflammasome and downstream IL-1β could improve clinical outcomes in patients with COVID-19.^18^ Indeed, European Medicines Agency (EMA) recommended anakinra (a recombinant human IL-1 receptor antagonist) for COVID-19 in adults with pneumonia requiring supplemental oxygen and who are at risk of developing severe respiratory failure, as determined by blood levels of a protein called soluble urokinase plasminogen activator receptor (suPAR) of ≥6 ng/mL.^19^

In this review, we summarize current evidence supporting the involvement of the NLRP3 inflammasome and IL-1β in the pathogenesis of COVID-19. We also discuss the therapeutic relevance of targeting IL-1β to improve COVID-19-related outcomes.

## Search strategy and selection criteria

Search strategy and selection criteria for references for the present Review were identified through PubMed using the following search terms and their combination: “SARS-CoV-2”, “pneumonia”, “COVID-19”, “ARDS”, “hyperinflammation”, “cytokine storm”, “NLRP3”, “inflammasome”, “IL-1” from December, 2019 until April, 2022. Only papers published in English were reviewed and considered. Additional papers identified from the reference list of the retrieved articles were also considered. The final reference list was generated on the basis of originality and relevance to the broad scope of this Review.

## Overview of the IL-1 signaling pathway

The IL-1 cytokine family comprises 11 members that can bind to 10 receptors.^20^ IL-1 cytokines can be either pro-inflammatory or anti-inflammatory, and their production and biological activity is finely modulated by numerous mechanisms.^20^ IL-1 receptor antagonist (IL-1Ra), IL-36Ra, IL-37, and IL-38 exhibit anti-inflammatory properties, with the former two being specific for their receptors.^20^ In addition, the extracellular domains of these receptors can be cleaved and released into the circulation to blunt inflammation. Typical pro-inflammatory members are IL-1α, IL-1β, and IL-18. IL-1α production may be induced in myeloid cells, however its precursor is constitutively present in all mesenchymal cells.^20^ During tissue stress or damage, a wide range of stimuli including local hypoxia, reactive oxygen species (ROS), extracellular ATP and danger-associate molecular patterns (DAMPs) promotes, though nuclear factor κB (NF-κB), the transcription and subsequent translation of pro-inflammatory genes including those coding for pro-IL-1α, pro-IL-1β and pro-IL-18 as well as the components of the NLRP3 inflammasome.^20^ IL-1β is an inducible cytokine mainly secreted by monocytes, macrophages and neutrophils.^17^ Both IL-1α and IL-1β remain within the cell when synthesized as they lack a signal peptide. Pro-IL-1β requires processing to mature IL-1β by caspase-1, whereas pro-IL-1α is already active, although it can be processed by several enzymes that increase its biological activity. Besides exerting pro-inflammatory effects, IL-1α can also act as an ‘alarmin’ and sustains IL-1β production.^7^

**Figure 1:**
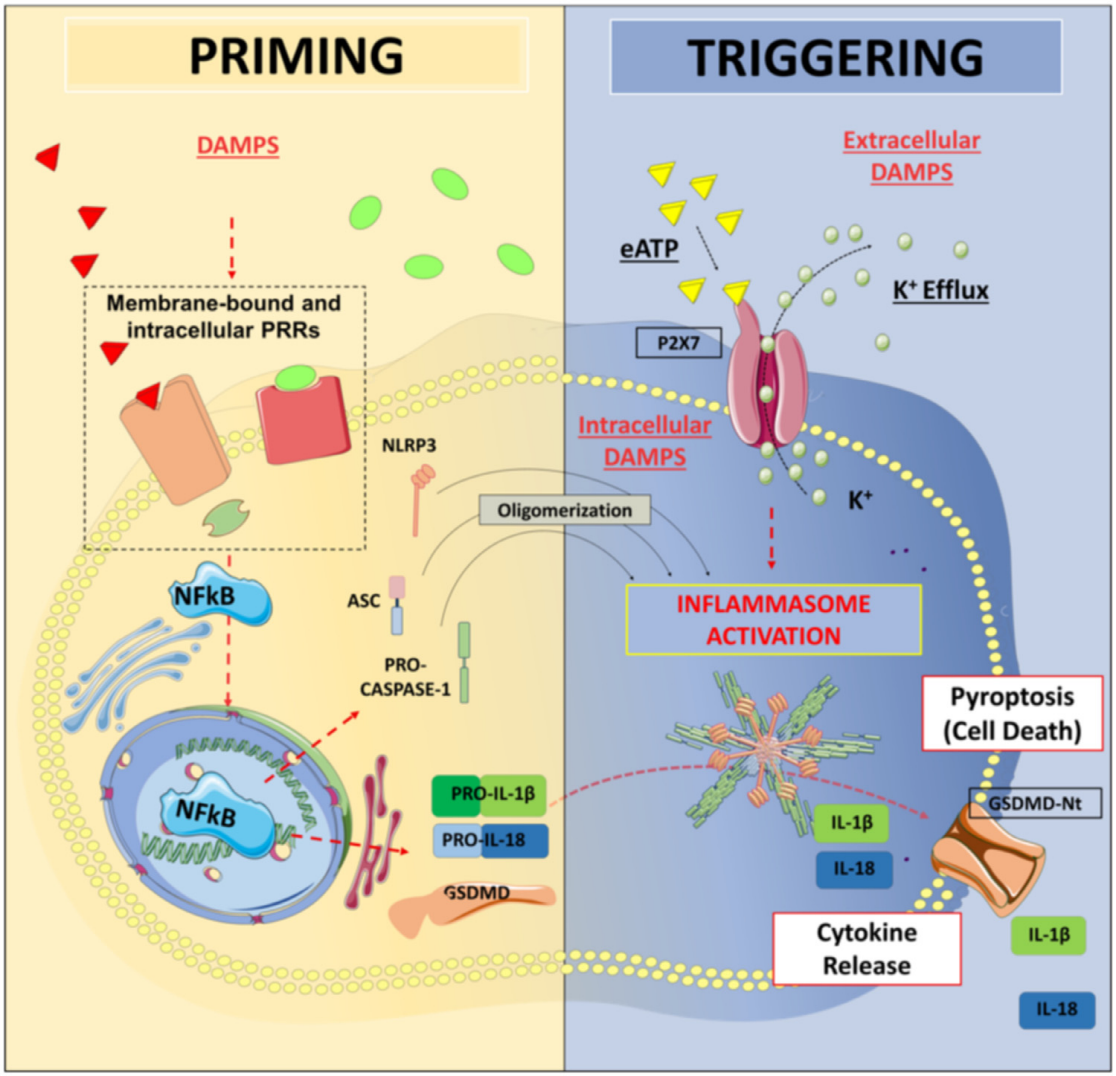
**Regulation of the NLRP3 inflammasome.** The activation of the NLRP3 inflammasome relies on two independent steps. The first one is a priming step triggered by DAMPs and leads to the transcription and translation of pro-inflammatory genes, including those of pro-IL-1β and IL-18 and the components of the NLRP3 inflammasome. The second signal triggers the activation of the inflammasome, that relies on the oligomerization of the three components. Following the autocatalytic cleavage of pro-caspase-1 to active caspase-1, this enzyme cleaves pro-IL-1β to its active form and forms the gasdermin channel for the release of IL-1β and IL-18 from the cell. Legend. DAMPs: damage-associated molecular patterns. eATP: extra-cellular ATP. GSDMD: gasdermin D. GSDMD-Nt: N-terminal fragment of gasdermin D. IL: interleukin. K^+^: potassium. NF-kB: nuclear factor κB. NLRP3: NACHT, leucine-rich repeat, and pyrin domain-containing protein 3. PRR: pattern recognition receptor. Reproduced with permission from “NLRP3 Inflammasome in Acute Myocardial Infarction” by Mauro et al. .^124^

IL-1-induced signalling is mediated by IL-1 receptor type 1 (IL-1R1, the ligand-binding chain) and its co-receptor IL-1R3. When IL-1α or IL-1β bind to IL-1R1, a structural change occurs so that IL-1R3 interacts with IL-1R1 forming a heterotrimeric complex thereby initiating intracellular signalling. The intracellular portions of IL-1R1 and IL-1R3 contain the Toll/IL-1-receptor (TIR) domain which is analogous to the TIR domain of toll-like receptors (TLRs) associated with pathogens. When the TIR domains of IL-1R1 and IL-1R3 are close, the adaptor protein myeloid differentiation factor 88 (MyD88) binds TIR domains and is responsible for a number of phosphorylations resulting in the activation of NF-κB.^20^ Pro-IL-1β is processed into mature IL-1β through caspase-1 activated by the inflammasome within the cell. Activation of the NLRP3 inflammasome may require two signals in macrophages ^17^^,^^21^ (Figure 1). However, circulating monocytes release active IL-1β after a single stimulation of the TLR, resulting from constitutively activated caspase-1 and release of endogenous adenosine triphosphate (ATP). In addition, pro-IL-1β released at the site of injury may be processed extracellularly by neutrophil proteolytic enzymes irrespective of NLRP3 and caspase-1.^22^ More details on IL-1 cytokine family and their roles in health and disease have been reviewed elsewhere.^17^^,^^20^

## IL-1 blockade across a wide spectrum of diseases

The growing knowledge regarding the pathophysiological role of IL-1 across a wide spectrum of cardiovascular and non-cardiovascular diseases has led to the investigation of different strategies to inhibit IL-1 signalling (Supplementary Table 1). Blockade of IL‑1 signalling has provided evidence about the role of inflammation in diseases where inflammation was not initially regarded to play a role (i.e. HF, atherosclerosis, diabetes), thus paving the way to novel therapeutic strategies.^23^

Three pharmacological agents targeting IL-1 are currently available: anakinra, canakinumab and rilonacept. Anakinra is a recombinant human IL-1 receptor antagonist approved by Food and Drug Administration (FDA) for moderate-to-severe active rheumatoid arthritis, neonatal-onset multisystem inflammatory disease, and deficiency of IL-1 receptor antagonist (DIRA). EMA approved anakinra also for Still's disease. However, anakinra is often used for other indications, including autoinflammatory diseases,^24^ recurrent pericarditis refractory to first-line agents,^25^ and gout flares in patients with contraindications to conventional therapies.^26^ Canakinumab is a human anti-IL-1β monoclonal antibody approved by FDA for cryopyrin-associated periodic syndromes (CAPS), tumour necrosis factor receptor-associated periodic syndrome (TRAPS), hyperimmunoglobulin D syndrome (HIDS)/mevalonate kinase deficiency (MKD), familial Mediterranean fever (FMF), and Still's disease. EMA extended its use also for symptomatic treatment of adult patients with frequent gouty arthritis. Rilonacept is a dimeric fusion protein combining two IL-1 receptors with an Fc immunoglobulin tail, usually referred to as ‘IL-1 trap’ as it can bind both IL-1α and IL-1β. Rilonacept has been FDA-approved for CAPS and, recently, for recurrent pericarditis.^27^ Of note, rilonacept is no longer approved by the EMA as the marketing authorization for rilonacept has been withdrawn at the request of the marketing authorization holder. Other indications for which rilonacept may be considered are systemic juvenile idiopathic arthritis, sJIA, and DIRA.

## IL-1 signaling in other highly pathogenic coronavirus infections

The NLRP3 inflammasome was described to be activated by a number of highly pathogenic human coronaviruses, like SARS-CoV and MERS-CoV.^12^^,^^28^^,^^29^ Different mechanisms accounting for NLRP3 inflammasome activation were described. Highly pathogenic human coronaviruses present transmembrane viral proteins acting as ion channels known as viroporins, such as open reading frame 3a (ORF3a) and SARS-CoV envelope (E) protein.^12^ SARS-CoV ORF3a activity is requiredfor NLRP3 inflammasome-mediated IL-1β release, with potassium efflux and mitochondrial ROS playing a central role in this process.^10^ ORF3a was also found to induce pro-IL-1β transcription through activation of NF-κB, that is triggered by tumor necrosis factor receptor-associated factor 3 (TRAF3)-mediated ubiquitination of p105 and apoptosis-associated speck-like protein (ASC).^30^ SARS-CoV-2 ORF3a has also been found to activate the NLRP3 inflammasome thus favouring IL-1β maturation and activation of of gasdermin D (GSDMD).^31^ SARS-CoV E protein inserts into the endoplasmic reticulum Golgi intermediate compartment allowing calcium flow from the cytosol, that is the main trigger for the activation of the NLRP3 inflammasome then increasing IL-1β production.^11^^,^^32^ In macrophages, ORF8b activates the NLRP3 inflammasome by providing a potent signal 2 for activation. In particular, it interacts with the leucine-rich repeat domain of the NLRP3 and localizes with NLRP3 and ASC.^9^ In addition, ORF8b is able to stimulate cell death through pyroptosis.^9^ Importantly, E protein and ORF3a are present also in SARS-CoV-2 and show a high amino acid identity compared with other human coronaviruses differently from ORF8b that was not conserved.^2^^,^^33^ While the activity of SARS-CoV-2 ORF3a and SARS-CoV-2 E protein was confirmed to participate to NLRP3 inflammasome activation, additional mechanisms have been described and will be discussed later. For example, SARS-CoV- nucleocapsid protein can bind NLRP3 to induce inflammasome oligomerization and IL-1β release in macrophages and dendritic cells.^34^

## IL-1 as a driver of hyperinflammation and damage in COVID-19

Following viral infections, timely and efficient activation of the innate immune system is a crucial step to limit viral replication/spread and tissue damage, stimulate acquired immunity with antibody production, and promote viral clearance and tissue healing.^35^ Conversely, excessive inflammation resulting from a dysregulated innate immune response exacerbates tissue damage and is associated with poor clinical outcomes.^35^ Due to its highly pro-inflammatory and pleiotropic biological activity, IL-1β plays a central role in orchestrating the innate immune responses following sterile and non-sterile injury across a wide range of conditions.^20^^,^^21^^,^^36^^,^^37^ The link between dysregulated IL-1β release and infections is well substantiated, and it has been demonstrated for several viruses, including SARS-CoV and MERS-CoV.^8^, ^9^, ^10^, ^11^^,^^30^^,^^38^

Since the outbreak of COVID-19, initial reports from China outlined the importance of the host immunity in determining disease evolution.^39^^,^^40^ Further studies confirmed uncontrolled and prolonged production of pro-inflammatory cytokines by monocytes and macrophages in response to SARS-CoV-2 which may result in reduced number of circulating cluster of differentiation (CD)4^+^/CD8^+^ T cells. Indeed, lymphocytopenia is one of the key immunological features of severe COVID-19.^41^^,^^42^ Recent findings point to a contribution of classical pro-inflammatory monocytes and hyperactivated neutrophils in triggering a myeloid-driven atypical cytokine storm, i.e. different from macrophage activation syndrome especially for (i) the markedly reduced type II interferon signaling and (ii) the vascular remodelling suggested by elevated systemic vascular endothelial growth factor levels.^43^ Histopathological analyses of lungs of patients deceased from COVID-19 show diffuse alveolar damage with pneumocyte desquamation, fibrin deposits, hyaline membranes, endothelial injury, capillary microthrombi, together with abundant exudative inflammation and massive infiltration of macrophages and CD4^+^/CD8^+^ T cells,^4^^,^^41^^,^^44^, ^45^, ^46^ that are expression of immunothrombosis and microcoagulopathy.^4^^,^^47^ Indeed, besides lymphocytopenia, several inflammatory biomarkers including C-reactive protein (CRP) and numerous cytokines (IL-1, IL-4, IL-6, IL-8, IL-17A), monocyte chemoattractant protein-1 (MCP-1), tumour necrosis factor (TNF), and interferon gamma (IFN-γ), are significantly upregulated in patients with severe disease manifestations and poor prognosis.^41^^,^^42^^,^^48^^,^^49^ Nevertheless, the role of these cytokines in the pathogenesis of COVID-19 remains unclear.

**Figure 2:**
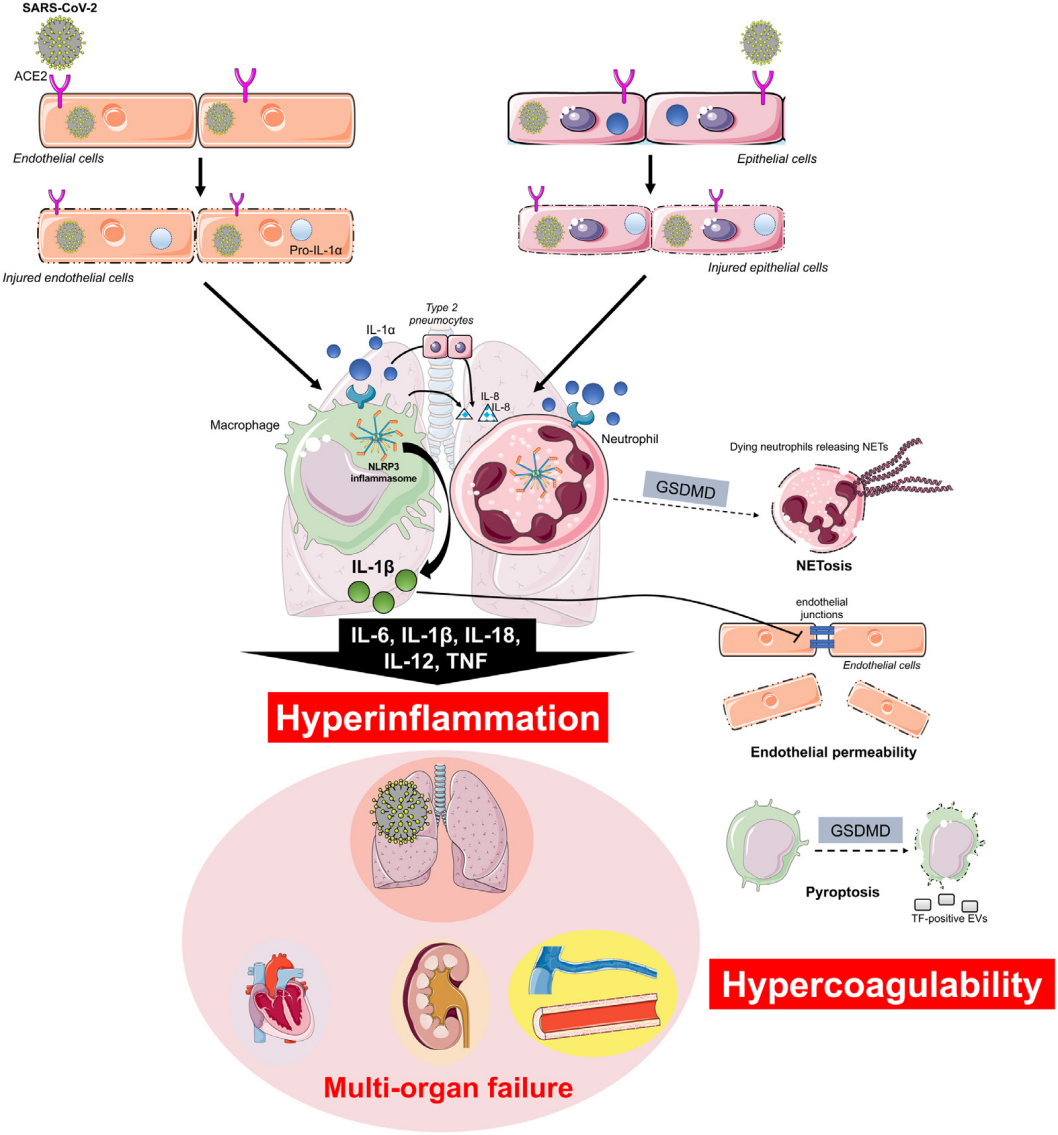
**General concepts about SARS-CoV-2 infection.** SARS-CoV-2 initiates after the virus entry into epithelial or endothelial cells of the lung through the ACE2 receptor. Within the cell, the virus replicates and causes cell injury and death. While dying, these cells release their content, particularly the pro-form of IL-1α. This is a pro-inflammatory cytokine which is responsible for the early phases of inflammation. Following this stage, immune cells infiltrate the lung – namely resident macrophages, neutrophils, and dendritic cells, that present antigens to T-cells in the lymph nodes. Here, B-cells are responsible for the production of specific antibodies against SARS-CoV-2, that allow for viral clearance in a few days. Once pro-IL-1α is locally released, it binds IL-1 receptor that is abundant in epithelial and endothelial cells of the lung. Pro-IL-1α is active without processing and triggers type 2 pneumocytes to release IL-8, thus increasing the number of infiltrating neutrophils into the lung. The neutrophil-mediated damage of pulmonary cells is responsible for early respiratory symptoms. IL-1α is also responsible for triggering IL-1β and G-CSF release from macrophages. IL-1β and G-CSF stimulate the bone marrow to release neutrophils and monocytes into the circulation and reach the lungs. Here, they interact with the endothelium, open endothelial junctions, increase the infiltration of immune cells, and release a wealth of pro-inflammatory cytokines (IL-6, IL-1β, IL-18, IL-12, TNF). After being processed by the NLRP3 inflammasome, IL-1β triggers IL-6 release that stimulate the liver to produce high levels of CRP, ferritin, and D-dimer, that are considered markers of COVID-19. Due to increased inflammatory burden, any organ other than the lungs can be involved and COVID-19 may progress toward multi-organ failure. In parallel, NETosis, endothelial dysfunction and tissue factor-positive extracellular vesicles released by monocytes following pyroptosis contribute to activate the coagulation cascade and promote hypercoagulability. The latter synergizes with hyperinflammation ending up in the process called immunothrombosis, that sustains multi-organ failure. Legend. ACE2: angiotensin-converting enzyme 2. EV: extracellular vesicle. GSDMD: gasdermin D. IL-1α: interleukin-1α. IL-1β: interleukin-1β. IL-8: interleukin-8. NETs: neutrophil extracellular traps. NLRP3: NACHT, leucine-rich repeat, and pyrin domain-containing protein 3. SARS-CoV-2: severe acute respiratory syndrome coronavirus 2. TF: tissue factor. This figure has been created using Servier Medical Art templates, which are licensed under a Creative Commons Attribution 3.0 Unported License; https://smart.servier.com.

**Figure 3:**
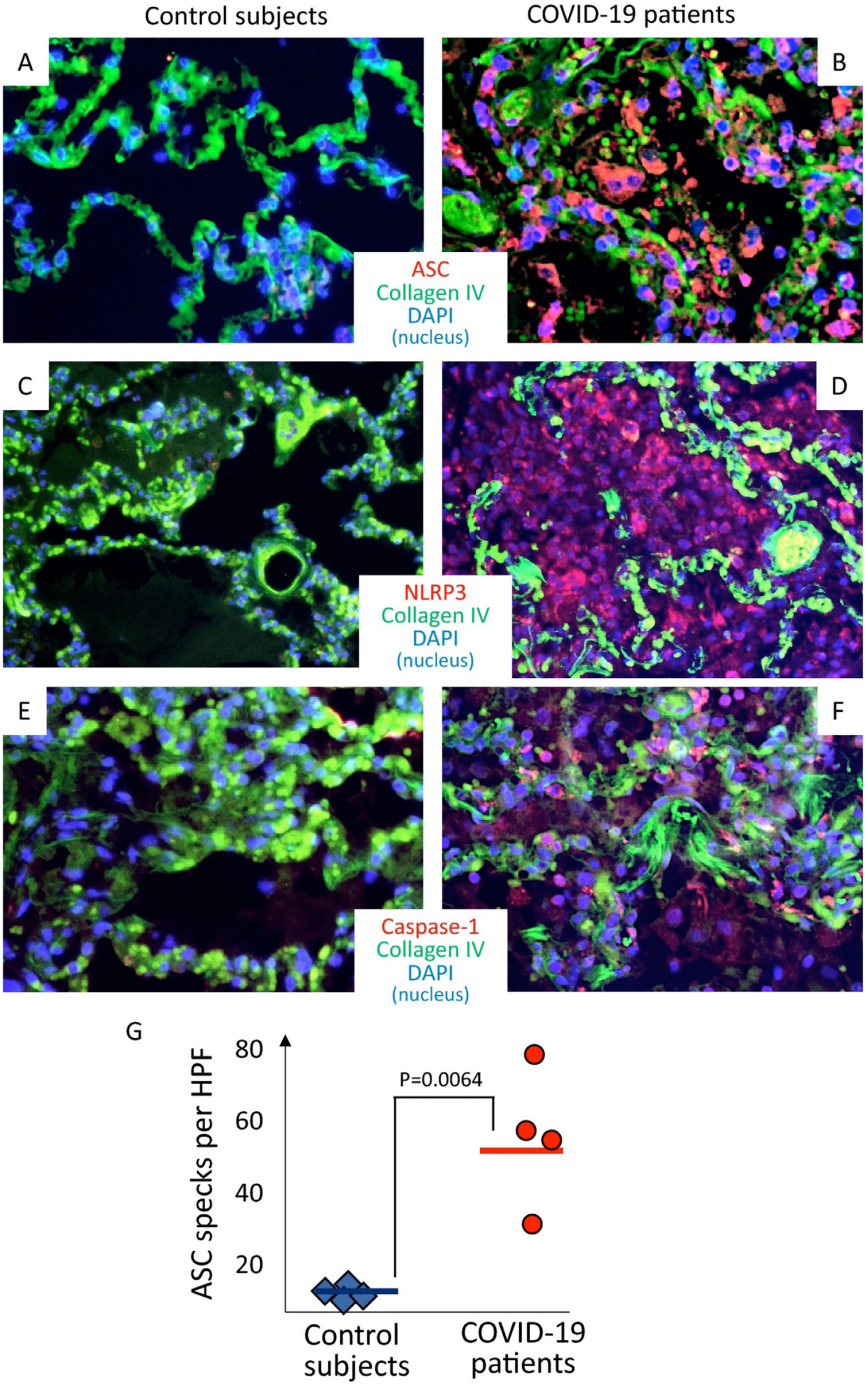
**NLRP3 inflammasome activation in the lungs of COVID-19 patients who died.** Immunofluorescence stainings from patients with fatal COVID-19 (**panels a, c, and e**) and from controls – individuals who suffered from cardiopulmonary arrest without evidence of lung infection (**panels b, d and f**) are provided. A significantly higher number of ASC specks in COVID-19 patients compared with controls in panel **g**. Legend. ASC: apoptosis-associated speck-like protein containing a caspase recruitment domain. NLRP3: NACHT, leucine-rich repeat, and pyrin domain-containing protein 3. Reproduced with permission from “Inflammasome formation in the lungs of patients with fatal COVID-19” by Toldo et al.^16^

As evidence was mounting, a pathogenetic role for the NLRP3 inflammasome/IL-1β signalling pathway in COVID-19 was established (Figure 2). Higher serum levels of IL-1β are associated with increased COVID-19 severity, and may allow the identification of patients at higher risk for poor clinical outcomes.^50^ In addition to IL-1β, circulating levels of IL-1Ra, IL-18 and IL-18 binding protein (IL-18BP) are increased in hospitalized COVID-19 patients.^15^^,^^50^ Caspase-1 and IL-18 positively correlated with IL-6 and CRP, with IL-18 levels measured at hospital admission correlating with progression to mechanical ventilation.^13^ Peripheral blood mononuclear cells (PBMCs) derived from severely ill COVID-19 patients display elevated expression of highly active NLRP3 inflammasome, as reflected by abundant levels of cleaved caspase-1 as well as mature IL-1β and IL-18 released upon stimulation *in vitro*.^42^ Interestingly, large IL-1β secretion upon stimulation has also been reported in macrophages derived from outpatients with COVID-19, suggesting that innate immunity dysregulation may also occur in mild disease or asymptomatic infection.^51^ Single-cell profiling of bronchoalveolar lavages from critically ill COVID-19 patients revealed the abundant presence of inflammatory IL-1β-secreting myeloid cells.^52^ Consistent with these observations, intense expression of NLRP3 inflammasome aggregates was detected, primarily in leukocytes, within areas of lung injury in patients with fatal COVID-19 pneumonia, providing further evidence on the molecular link between SARS-CoV-2 infection, IL-1-driven hyperinflammation, and pulmonary damage (Figure 3).^16^

Multiple studies have demonstrated that the interaction of SARS-CoV-2 with ACE2 receptor triggers the NLRP3 inflammasome with subsequent release of IL-1β and other inflammatory cytokines, which lead to hyperinflammation, inflammatory cell death and diffuse organ damage^53^^,^^54^ (Figure 3). Exposure of primary human monocytes to SARS-CoV-2 single-stranded ribonucleic acid (RNA) rapidly triggers NLRP3 inflammasome assembly and IL-1β processing and release. On the contrary, NLRP3 silencing or caspase-1 pharmacological inhibition completely abolishes IL-1β production.^55^ In this experimental setting, viral RNA induces mature IL-1β release through a non-classical pathway dependent on caspase-1, caspase-8, NLRP3, potassium efflux and autophagy, and independent of GSDMD processing and pyroptosis.^55^ In infected human monocytes, the nucleocapsid protein of SARS-CoV-2 was shown to associate with GSDMD and inhibit GSDMD processing by caspase-1, thereby antagonizing IL-1β secretion and pyroptosis of the host cell.^56^ Nevertheless, Ferreira et al*.* reported that SARS-CoV-2-infected human monocytes may undergo, upon NLRP3/caspase-1 activation and GSDMD cleavage, lytic cell death by pyroptosis, which was prevented by the administration of the NLRP3 inhibitor glyburide.^57^ While the magnitude of the effect of SARS-CoV-2 on host pyroptosis remains unclear, the discrepancy in the aforementioned observations may be due to differences in the experimental setting, as the exposure to different viral antigens or the viable virus may elicit distinct cellular events.^53^, ^54^, ^55^^,^^57^ Olajide et al. have found that stimulation of PBMCs with recombinant SARS-CoV-2 spike protein S1 resulted in NF-kB translocation to the nucleus, NLRP3/caspase-1 expression, and activation with subsequent production of IL-1β, IL-6, IL-8, and TNF.^58^ Importantly, treatment with BAY11-7082, an NF-kB inhibitor, or MCC950, a selective NLRP3 inhibitor, significantly reduced IL-1β release, while dexamethasone reduced IL-6 without affecting IL-1β levels.^58^ Rodrigues *et al.* showed that infection of PBMCs derived from healthy subjects with viable SARS-CoV-2 induces the formation of NLRP3 and ASC puncta and caspase-1 activation, with subsequent release of IL-1β in the media.^13^ In line with other observations, the NLRP3 inhibitor MCC950 abolished SARS-CoV-2-induced NLRP3 assembly, caspase-1 activation and IL-1β production.^13^ Analyses of PBMCs from COVID-19 patients incubated with the NLRP3 trigger nigericin revealed that the activation potential of the NLRP3 inflammasome varies across myeloid cell subsets and COVID-19 severity.^50^ While the nigericin-triggered NLRP3 activation in intermediate and classical monocytes was comparable to that of uninfected subjects, it was specifically increased in CD14^dim^CD16^+^ nonclassical monocytes, and inversely correlated with the decreased number of these cells in severe-to-critical COVID-19 patients. On the other hand, CD66b^+^CD16^dim^ granulocytes displayed decreased NLRP3 activation potential, which correlated with disease severity.^50^ These findings have suggested that the inflammasome response in myeloid cells may serve as a biomarker of COVID-19 evolution, and point out the presence of a cell- and disease severity-specific NLRP3 signature.^50^ Theobald *et al.* have reported excessive macrophage NLRP3 activation and IL-1β release following *in vitro* stimulation with nigericin or ATP also in patients with only mild disease compared to SARS-CoV-2 naïve individuals. This opens the possibility of a quantitative rather than qualitative effect driving COVID-19-associated hyperinflammation.^51^ Multiple studies have reported that the inflammatory immune signature involving the NLRP3 inflammasome and IL-1β extends for several weeks after recovery from COVID-19.^42^^,^^51^^,^^59^ These observations may suggest that persistent inflammasome activation, and possibly prolonged detection of viral RNA in some patients after convalescence, may potentially increase susceptibility for long COVID including pulmonary, neurological and cardiovascular manifestations.^42^^,^^51^^,^^59^^,^^60^ In parallel, these observations have suggested that the NLRP3 inflammasome and IL-1β may aid the innate immune memory after recovery from COVID-19 and contribute to the immunogenicity of SARS-CoV-2 vaccines.^51^

In addition to its role in leukocytes, the NLRP3 inflammasome/IL-1β signalling pathway regulates the biological responses of several other cell types. After exposure to SARS-CoV-2, uncontrolled NLRP3 activation, IL-1β release, and pyroptosis have been demonstrated in human hematopoietic stem/progenitor cells and in endothelial progenitor cells, with the effects being significantly reduced by inhibition of NLRP3 signalling.^53^^,^^54^ Therefore, SARS-CoV-2, through the NLRP3 inflammasome and IL-1β, may also directly impair haematopoiesis and the proliferative potential of the vascular endothelium, with potentially detrimental consequences in the short- and long-term.^53^ Furthermore, the hyperinflammatory state associated with COVID-19 contributes to hypercoagulability, eventually leading to macrovascular (arterial, venous) and microvascular thrombosis, which are frequent in hospitalized COVID-19 patients and account for considerable organ damage and mortality.^49^^,^^61^, ^62^, ^63^, ^64^ Several pro-inflammatory cytokines including as IL-1α, IL-1β and downstream IL-6 are known to exert multiple pro-thrombotic effects. IL-1β and IL-6 were previously shown to induce, even at low concentrations, significant viscoelastic and ultrastructural modifications in platelets and erythrocytes, and to enhance coagulability of whole blood.^65^ IL-1α and IL-1β can act as potent inducers of platelet activation and aggregation, and megakaryocyte maturation. In addition, exposure of endothelial cells to IL-1α or IL-1β considerably enhances the expression of adhesion molecules (e.g., P-selectin, vascular cell adhesion molecule-1 [VCAM-1], intercellular adhesion molecule-1 [ICAM-1]) and pro-coagulant molecules (e.g., tissue factor [TF], von Willebrand factor [VWF]), and downregulates anticoagulant pathways (e.g., protein C, thrombomodulin). These events promote recruitment and subsequent activation of leukocytes and platelets at the site of vascular injury and stimulate coagulation.^66^^,^^67^ Accordingly, exosomes derived from patients with severe COVID-19 were found to trigger robust mRNA expression of NLRP3, caspase-1 and IL-1β across multiple endothelial cell lineages, thus suggesting that inflammasome activation and IL-1β release may contribute to the systemic endotheliopathy and coagulopathy associated with severe COVID-19.^68^ Once recruited, activated platelets themselves are able to release microparticles containing IL-1β, which increases endothelial permeability,^69^ and to interact with leukocytes to stimulate NLRP3 inflammasome transcription and activation in these cells leading to a feed-forward thromboinflammatory circuit.^70^ Inflammasome-mediated pyroptosis of macrophages can also induce the release of microvescicles rich in TF to initiate clotting.^4^ A strong bidirectional interplay between inflammasome activation and neutrophil extracellular trap (NET) formation (i.e. NETosis) exists.^71^^,^^72^ NETs, primarily generated by neutrophils, are extruded web-like structures composed of DNA filaments, histones and proteins with antimicrobial activity (e.g., neutrophil elastase, myeloperoxidase) that work to entrap and kill pathogens . However, excessive NETosis in the vasculature, as reported in patients with COVID-19, can contribute to pathological thrombosis (both arterial and venous) by providing a scaffold for platelets, erythrocytes, extracellular vesicles, and procoagulant molecules (e.g, VWF, TF), and by activating the intrinsic coagulation pathway (e.g., factor XII) and degrading both endogenous anticoagulant (e.g., TF pathway inhibitor) and fibrinolytic (tissue-type plasminogen activator) agents.^4^^,^^43^^,^^73^ The complement system has also been shown to contribute to hyperinflammation and thrombosis in severe COVID-19.^74^ Complement system activation was previously described to induce NLRP3 inflammasome activation and augment the secretion of both IL-1α and IL-1β.^75^ The intricated, yet unclear interplay between complement system and inflammasome pathway activation may therefore represent an additional molecular link between dysregulated immunity and pathological thrombosis in COVID-19.

Although evidence is limited, a role for IL-1α might be hypothesized (Figure 2). IL-1α release by epithelial cells is sensed by inflammatory myeloid cells, which leads to inflammasome activation and sustains the inflammatory cascade.^76^ Also endothelial cells may express IL-1α thus stimulating granulocyte recruitment and inflammatory thrombosis.^76^

In summary, several lines of evidence indicate that IL-1 and NLRP3 inflammasome are mechanistically involved in the pathogenesis of COVID-19.^12^^,^^77^ Hyperactivation of the NLRP3/IL-1β signalling pathway has been associated with adverse disease manifestations. It was therefore hypothesized that pharmaceutical interventions targeting this major pro-inflammatory signalling pathway may improve clinical outcomes in patients with COVID-19.

## Pharmacological blockade of IL-1 in COVID-19

As IL-1 plays a role in the pathogenesis of COVID-19, it is tempting to hypothesize that IL-1 blockade may result in an attenuated inflammatory response and improved clinical outcomes.^6^ A number of observational studies have provided promising results for anakinra in COVID-19 patients in terms of respiratory improvement, reduced need for invasive mechanical ventilation, and improved overall survival along with a rapid reduction in inflammatory markers (e.g. CRP), as compared to standard-of-care (SOC)^78^, ^79^, ^80^, ^81^, ^82^, ^83^ (Table 1). These findings were also supported by two meta-analyses of non-randomized cohort studies.^84^^,^^85^

**Table 1:** Observational studies investigating IL-1 blockers in COVID-19 patients.

	Anakinra		
*Authors*	*Patients*	*Drug regimen*	*Main findings*
Cavalli et al.^78^	52 patients with moderate-to-severe COVID-19: • 16 patients treated with SOC; • 7 treated with low-dose anakinra on top of SOC; • 29 treated with high-dose anakinra on top of SOC	Low-dose anakinra: 100 mg SC bid High-dose:10 mg/kg IV daily (5 mg/kg bid infused over 1 h) Duration of treatment was decided according to clinical benefit, or until death, bacteremia, or AE occurrence	High-dose anakinra reduced CRP levels and improved respiratory function after 21 days compared to SOC-treated patients. High-dose anakinra improved the survival rate at 21 days compared to SOC-treated patients (90% vs. 56%; HR 0.20, 95% CI 0.04–0.63, *p*=0.009). Most common AEs in high-dose anakinra-treated patients were bacteremia (14%) and increase in liver enzymes (10%). Low-dose anakinra-treated patients did not show any effect neither on CRP levels nor on clinical improvement after 7 days and was withdrawn.
Huet et al., Ana-COVID study^79^	96 patients with moderate-to-severe COVID-19: • 44 treated with SOC; • 52 treated with anakinra	Anakinra 100 mg SC bid for 72 h, then 100 mg SC daily for 7 days (adapted to renal function if needed)	Need for IMV or death occurred in 25% of patients in the anakinra group compared with 73% of SOC-treated patients (HR 0.22, 95% CI 0.11–0.41, *p*<0.001). This was confirmed also considering need for IMV or death alone (HR 0.22, 0.09-0.56, *p*=0.001 and HR 0.30, 95% CI 0.12–0.71, *p*=0.006, respectively). A significant decrease in CRP levels was persistently observed for anakinra-treated vs. SOC-treated patients starting from day 2 (*p*<0.001). Common AEs in the anakinra group were increase in liver aminotransferase (13% vs. 9%) and thromboembolic events (19% vs. 11%) vs. SOC-treated patients.
Cauchois et al.^80^	22 patients with moderate-to-severe COVID-19: • 10 treated with SOC; • 12 with anakinra on top of SOC (except for antivirals)	Anakinra: 300 mg IV daily for 5 days, then 200 mg IV daily for 2 days, and then 100 mg IV daily for 1 day	Patients treated with anakinra experienced less frequently need for IMV (17% vs. 40%). No patient in the anakinra died compared to 1 patient in the SOC group. Clinical improvement at day 5 was achieved more frequently for anakinra- compared to SOC-treated patients (*p*<0.01). Patients treated with anakinra showed a rapid reduction of fever within 48 h compared to SOC-treated patients (*p*<0.05).
Aouba et al.^81^	9 patients with moderate-to-severe COVID-19 treated with anakinra	Anakinra: 100 mg bid from day 1 to 3, then at 100 mg daily from day 4 to 10	Eight out of 9 patients showed a reduction in body temperature and inflammatory markers and an improvement in oxygenation after 3 days. In all patients, chest CT scan between day 5 and 8 showed no evolution of pulmonary lesions. No death was recorded.
Navarro-Millán et al.^82^	11 patients with severe COVID-19 treated with anakinra on top of SOC	Anakinra: 100 mg SC every 6 h, then decreased to every 8, 12, and 24 h over a maximal duration of 20 days (tapering based on clinical status)	Patients treated with anakinra within 36 h from enrolment did not require IMV. Three out of 4 patients needing IMV and treated with anakinra were successfully extubated. Ferritin and CRP dropped down or normalized during anakinra treatment in all but one patient. Bacterial infections and elevation of transaminases occurred in 3 patients.
Pontali et al.^83^	5 patients with moderate-to-severe COVID-19 treated with anakinra on top of SOC	Anakinra: 100 mg IV tid (300 mg/daily) for 24–48 h with tapering based on clinical status	All patients experienced rapid resolution of systemic inflammation and an improvement in respiratory parameters. No AEs were observed.
**Canakinumab**			
Ucciferri et al.^91^	20 patients with moderate-to-severe COVID-19: • 10 treated with SOC; • 10 treated with canakinumab on top of SOC	Canakinumab: 300 mg SC in a single dose	Canakinumab-treated patients experienced a rapid and significant reduction in CRP levels at day 1 and 3 and an increase in the PaO_2_/FiO_2_ ratio from baseline to day 3 and 7 post-treatment compared to SOC-treated patients. After 45 days, all patients were alive and discharged from hospital without need for oxygen therapy. Among SOC-treated patients, 1 patient died and 9 were discharged from hospital, with 1 of them needing oxygen therapy. No AE was recorded in canakinumab-treated patients.
Falasca et al.^92^	34 patients with moderate-to-severe COVID-19: • 17 treated with SOC • 17 treated with canakinumab on top of SOC	Canakinumab: 300 mg SC in a single dose	Patients treated with canakinumab experienced a reduction in inflammatory biomarkers and a rapid increase in PaO_2_/FiO_2_ ratio compared to SOC-treated patients.
Generali et al.^93^	48 patients with moderate COVID-19: • 15 treated with SOC • 33 treated with canakinumab on top of SOC	Canakinumab: 150 mg SC on day 1 and on day 7	A larger proportion of patients treated with canakinumab was discharged within 21 days compared with SOC-treated patients (63% vs. 0%). Clinical improvement was observed after administration of canakinumab compared with SOC. Survival at 60 days was higher in patients treated with canakinumab than in patients treated with SOC (90% vs. 73.3%). Mild AEs were seen in patients treated with canakinumab.
Landi et al., CANASCOV study^94^	88 patients with moderate COVID-19 treated with canakinumab	Canakinumab: 300 mg SC in a single dose	Canakinumab improved respiratory function (PaO_2_/FiO_2_ ratio from 160, IQR 122-210 to 237, IQR 158-331; *p* <0.0001) after 7 days. A substantial reduction in CRP was observed (from 31.5 to 5.8 mg/L, *p* <0.0001). After 7 days, 58% of patients were discharged and 13.6% died. Overall survival at 1 month was 79.5%.
**Colchicine**			
Scarsi et al.^99^ Piantoni et al.^105^	262 patients with moderate-to-severe COVID-19: • 140 treated with SOC • 122 treated with SOC+colchicine	Colchicine 1 mg once daily or 0.5 mg once daily in case of severe diarrhoea	A reduced proportion of patients died among those treated with colchicine on top of SOC vs. SOC alone (16% vs. 37%, *p*<0.001). Treatment with colchicine was independently associated with a lower mortality risk (HR 0.15, 95% CI 0.06–0.37, *p*<0.0001). Long-term survival (i.e. after 270 days) was improved in patients treated with colchicine (20% vs. 44%, *p*=0.0001).
Sandhu et al*.*^104^	112 patients with moderate-to-severe COVID-19: • 78 treated with SOC • 34 treated with SOC+colchicine	Colchicine 0.6 mg twice daily for 3 days, then 0.6 mg once daily for 12 days; discontinued if discharge occurred before completing 15 doses	Patients on colchicine therapy experienced a reduced rate of death (47% vs. 81%, *p*<0.001) and intubation (47% vs. 87%, *p*<0.001) and an increased discharge rate (53% vs. 19%, *p*<0.001) compared to those treated with SOC alone.
Brunetti et al.^100^	66 patients with moderate-to-severe COVID-19: • 33 treated with SOC • 33 treated with SOC+colchicine	Not specified	Death for any cause within 28 days occurred less frequently in patients treated with colchicine vs. SOC (OR 0.20, 95% CI 0.05–0.80, *p*=0.023). On day 28, patients treated with colchicine showed a larger improvement in the WHO OSCI score (OR 3.50, 95% CI 1.19–10.28, *p*=0.023). A larger number of patients were discharged home on day 28 in the colchicine group vs. SOC (OR 5.0, 95% CI 1.25-20.08, *p*=0.023).
Pinzón et al.^103^	240 patients with moderate-to-severe COVID-19: • 95 treated with glucocorticoids • 145 treated with glucocorticoids+colchicine	Colchicine 0.5 mg bid for 7 to 14 days	No statistically significant difference was observed in patients treated with colchicine+glucocorticoids vs. glucocorticoids alone (9.6 vs. 14.6%, *p*=0.179).
Manenti et al.^102^	141 patients with moderate-to-severe patients: • 71 patients treated with SOC • 70 patients treated with SOC+colchicine	Colchicine 1 mg once daily from day 1 up to clinical improvement or to a maximum of 21 days Colchicine 0.5 mg once daily in case of severe diarrhoea or eGFR <30 mL/min/1.73 m^2^ Colchicine 0.5 mg once every other day in case of hemodialysis or liver impairment	Cumulative incidence of death after 3 weeks was lower in patients treated with colchicine vs. SOC (adjusted HR 0.24, 95% CI 0.09–0.67). Clinical improvement across three weeks occurred more frequently in colchicine-treated patients (adjusted RR 1.80, 95% CI 1.00–3.22). Skin rash and diarrhoea were the most common AEs in colchicine-treated patients.
Della-Torre et al.^101^	9 patients with COVID-19 treated at home with a loading dose of 1 mg colchicine PO bid followed by 1 mg daily until the 3^rd^ day of axillary temperature <37.5 °C		Colchicine was initiated after 8 (6-13) days from COVID-19 onset and after 3–5 days of spiking fever resistant to acetaminophen or antibiotic treatment. Body temperature dropped down to normal after 72 h in all patients. One patient was hospitalized due to persisting dyspnea and discharged 4 days later with home oxygen. Two patients complained about a mild diarrhoea that did not stop them from completing the whole treatment.

Abbreviations

AE: adverse event. aHR: adjusted hazard ratio. ARDS: acute respiratory distress syndrome. bid: bis in die. BMI: body mass index. CANASCOV: CANAkinumab Study on CoronaVirus pneumonia. CFR: case fatality rate. CI: confidence interval. COVID-19: coronavirus disease 2019. CRP: C-reactive protein. CPAP: continuous positive air pressure. CSS: cytokine storm syndrome. CT: computed tomography. CXR: chest X-ray. GI: gastrointestinal. HR: hazard ratio. hs-CRP: high-sensitivity C-reactive protein. ICU: intensive care unit. IL-6: interleukin-6. IV: intravenous(ly). IMV: invasive mechanical ventilation. OR: odds ratio. OSCI: ordinal scale for clinical improvement. PaO_2_/FiO_2_: ratio of arterial oxygen partial pressure to fractional inspired oxygen. PO: per os. RCT: randomized clinical trial. RR: risk ratio. SAE: severe adverse event. SC: subcutaneous(ly). SOC: standard-of-care. tid: ter in die. VAP: ventilation-associated pneumonia.

Anakinra has also been tested in randomized clinical trials (RCTs)^86^, ^87^, ^88^, ^89^ (Table 2). In two early RCTs – the CORIMUNO-ANA-1 (Cohort Multiple Randomized Controlled Trials Open-label of Immune Modulatory Drugs and Other Treatments in COVID-19 Patients-Anakinra trial) and REMAP-CAP (Randomized Embedded Multifactorial Adaptive Platform for Community-acquired Pneumonia) trials, treatment with anakinra did not show statistically significant benefits.^87^^,^^88^ The ANACONDA (Anakinra for COVID-19 Respiratory Symptoms) trial was a multicentre, open-label, randomized, controlled superiority trial comparing the efficacy of anakinra on top of SOC in hospitalized COVID-19 patients (NCT04364009) and was early terminated. The SuPAR-guided Anakinra treatment for Validation of the risk and Early management of severe respiratory failure by COVID-19 (SAVE) trial^90^ and the SuPAR-guided Anakinra treatment for Validation of the risk and Early Management Of seveRE respiratory failure by COVID-19 (SAVE-MORE) trial^86^ showed that anakinra shortened hospital stay, improved clinical status and reduced 28-day mortality (hazard ratio 0.45, *p*=0.045, as compared with placebo) when patients were stratified according to the levels of the inflammatory biomarkers. In light of the positive results of the SAVE-MORE trial, EMA recommended the extension of anakinra in adult COVID-19 patients at risk to develop severe respiratory failure, as determined by suPAR plasma levels ≥6 ng/ml, requiring supplemental oxygen.^19^

**Table 2:** Randomized clinical studies investigating IL-1 and NLRP3 inflammasome blockers in COVID-19 patients.

	Anakinra		
*Authors*	*Patients*	*Drug regimen*	*Main findings*
Kyriazopoulou et al., SAVE-MORE trial^86^	594 patients with COVID-19 at risk of progressing to respiratory failure (plasma suPAR ≥6 ng/mL): • 189 treated with SOC + placebo • 405 treated with SOC + anakinra	Anakinra: - 100 mg/day SC for 7–10 days 510 (85.9%) patients receiving dexamethasone	A large proportion of patients receiving anakinra showed a complete recovery with no viral RNA detected after 28 days compared with placebo (50.4% vs. 26.5%; adjusted proportional odds of a worse score on the 11-point WHO-CPS at day 28 with anakinra 0.36, 95% CI 0.26–0.50, *p* <0.0001 vs. placebo). Anakinra was independently associated with clinical benefit at day 14 compared with placebo (OR 0.58, 95% CI 0.42–0.79, *p*=0.001). In addition, treatment with anakinra significantly reduced the risk of persistent disease (OR of WHO-CPS ≥1, 0.36; 95% CI 0.25–0.53, *p* <0.0001) and severe disease or death at day 28 vs. placebo (OR of WHO-CPS ≥6, 0.46; 95% CI 0.26–0.83, *p*=0.010). A reduced number of patients treated with anakinra showed a progression toward severe respiratory failure or death by day 14 compared to placebo (20.7% vs. 31.7%; HR 0.62, 95% CI 0.45–0.87, *p*=0.005). In addition, anakinra reduced the risk of death by day 28 compared to placebo (3.2% vs. 6.9%; HR 0.45, 95% CI 0.21–0.98, *p*=0.045). Infections were the most frequent AE and occurred less frequently in the anakinra than in the placebo group (8.4% vs. 15.9%).
CORIMUNO-19 Collaborative group, CORIMUNO-ANA-1^87^	114 patients with mild-to-moderate COVID-19 pneumonia: • 55 treated with SOC • 59 treated with SOC + anakinra	Anakinra: - 200 mg IV bid on day 1 to 3, then 100 mg bid (total 200 mg) on day 4, and 100 mg once on day 5. - If no improvement on day 4, 3 supplementary days at 400 mg daily on day 4 to 6, followed by a decrease to 200 mg daily on day 7 and 100 mg daily on day 8, then treatment interruption.	A similar proportion in the anakinra and SOC arms showed a WHO-CPS score of >5 points at day 4 (36% vs. 38%). A reduced proportion of patients experiencing a significant event (non-IMV, HFO, IMV, or death) was observed in the anakinra arm compared to SOC (47% vs. 51%). No difference in the two groups was recorded for death at day 14, 28 and 90.
The REMAP-CAP Investigators, REMAP-CAP trial^88^	2216 patients with critical COVID-19 • 406 treated with SOC • 373 treated with anakinra • 1437 treated with other immunomodulators	Anakinra: - 300 mg IV as loading dose, followed by 100 mg every 6 h (12 h if CrCl <30 mL/min) for 14 days or until either free from IMV for more than 24 h, or discharge from ICU.	Median organ support-free days were similar for anakinra and SOC ( median adjusted OR 0.99, 95% CrI 0.74–1.35 vs. SOC). Hospital survival rates were similar between anakinra and SOC (60.3% vs. 63.1%; median adjusted OR 0.97, 95% CrI 0.66–1.40).
Declercq et al*.* COVAID^89^	342 patients with moderate-to-severe COVID-19 • 230 treated with SOC • 112 treated with SOC+anakinra	Anakinra: - 100 mg daily SC for 28 days or until hospital discharge - 100 mg every other day SC if eGFR < 30 mL/min/1.73 m^2^ 213 (62.3%) patients receiving glucocorticoids	No change in the median time to clinical improvement between groups was observed. No difference in mortality between treatment groups was found. Serious AEs were similar between study groups.
**Canakinumab**			
Caricchio et al. CAN-COVID trial ^95^	448 patients with severe COVID-19: • 223 treated with SOC + placebo • 225 treated with SOC + canakinumab	Canakinumab: 450 mg for body weight of 40–59 kg, 600 mg for 60–80 kg, or 750 mg for >80 kg in a single dose	The proportion of patients who survived without IMV from day 3 to day 29 was similar in the canakinumab and placebo group (88.8% and 85.7%, respectively; OR 1.39, 95% CI 0.76–2.54). The proportion of patients who died from COVID-19 by day 29 was lower in the canakinumab vs. placebo group (4.9% vs. 7.2%; OR 0.67, 95% CI 0.30–1.50). An exploratory analysis showed that less patients on canakinumab required IMV or rescue therapy with tocilizumab or anakinra compared to placebo (87.4% vs. 79.4%; OR 1.93, 95% CI 1.12–3.31). By day 29, more patients treated with canakinumab showed no clinical or virologic evidence of infection compared with placebo (35.2% vs. 30.4%). A reduced number of patients in the canakinumab arm was hospitalized on day 29 (5.3% vs. 8.4%).
**Colchicine**			
RECOVERY Collaborative Group RECOVERY Trial ^108^	11,340 patients with moderate-to-severe COVID-19: • 5,730 treated with SOC • 5,610 treated with colchicine	Colchicine: - 1 mg after randomization, then 0.5 mg 12 h later, followed by 0.5 mg bid for 10 days or until discharge, whichever came first, - or 0.5 mg once daily for patients receiving a moderate CYP3A4 inhibitor or with eGFR <30 mL/min/1.73 m^2^ or estimated body weight <70 kg	No difference in the proportion of patients who died for all causes was found in the two groups (1173 patients in the colchicine group vs. 1190 patients in the SOC group; RR 1.01, 95% CI 0.93–1.10, *p*=0.77). No secondary outcome (time to discharge, need for MV, or death) was met. Two AEs probably related to colchicine were recorded (AKI and rhabdomyolysis).
Deftereos et al. GRECCO-19 Randomized Clinical Trial ^106^	105 patients with moderate-to-severe COVID-19: • 50 treated with SOC • 55 treated with SOC+colchicine	Colchicine: - Loading dose 1.5 mg followed by 0.5 mg of colchicine after 1 h (loading dose 1 mg in case of azithromycin) Maintenance: dosage: 0.5 mg bid (once daily if body weight <60 kg) until hospital discharge or a maximum of 21 days	The proportion of patients treated with SOC experiencing a rapid clinical deterioration (based on WHO ordinal clinical scale) was higher in the SOC vs. colchicine group (14% vs. 1.8%; OR 0.11, 95% CI 0.01–0.96. *p*=0.046). Event-free 10-day survival was 83% vs. 97% in the control vs. interventional group (*p*=0.03). Diarrhoea was more frequent in the colchicine arm (45.5% vs. 18.0%, *p*=0.003).
Lopes et al*.*^107^	74 patients with moderate-to-severe COVID-19: • 37 treated with placebo • 37 treated with colchicine	Colchicine: - 0.5 mg tid for 5 days, then 0.5 mg bid for 5 days - If weight ≥80 kg, first dose was 1.0 mg - If eGFR <30 mL/min/1.73 m^2^, 0.25 mg tid for 5 days, then 0.25 mg bid for 5 days.	Patients treated with colchicine stopped using supplemental oxygen earlier than those in the placebo group (median 4 vs. 6.5 days, *p*<0.001) and had a shorter hospital stay (median 7 vs. 9 days, *p*=0.003). Across one week, patients treated with colchicine experienced a marked reduction in CRP levels compared with those in the placebo group (*p*<0.001). Diarrhoea and pneumonia occurred more frequently in the colchicine and placebo groups, respectively.
Tardif et al. ColCORONA Trial^109^	4,488 outpatients with mild-to-moderate COVID-19: • 2,253 treated with placebo • 2.235 treated with colchicine	Colchicine: - 0.5 mg bid for the first 3 days, then once daily for 27 days	No difference in the composite of death or hospitalization within 30 days from randomization (primary endpoint) was observed between groups. Among those with a PCR-confirmed COVID-19 diagnosis, the primary endpoint occurred less frequently in colchicine-treated patients vs. placebo (OR 0.75, 95% CI 0.57–0.99, *p*=0.04). The risk for hospitalization was decreased (OR 0.75, 95% CI 0.57–0.99), but no effect on death was seen. Serious AEs were 4.9% in the colchicine group and 6.3% in the placebo, with pneumonia occurring less frequent and diarrhoea more frequent in the colchicine arm.
Pascual-Figal et al. COL-COVID study^110^	103 patients with moderate-to-severe COVID-19: • 51 treated with SOC • 52 treated with SOC+colchicine	Colchicine: - Loading dose of 1.5 mg (1 mg followed by 0.5 mg 2 h later), then 0.5 mg bid for the next 7 days, and then 0.5 mg once daily until day 28	Colchicine significantly reduced the risk of clinical deterioration (OR 0.11, *p*=0.03). At day 28, all patients treated with colchicine were discharged alive, while 2 patients died in the standard treatment arm and one was still hospitalized.
Mareev et al. COLORIT study^111^	43 patients with moderate-to-severe COVID-19: • 22 treated with SOC • 21 treated with colchicine	Colchicine: 1 mg daily up to day 3, then 0.5 mg daily up to 14 days	The primary endpoint (SHOCS-COVID score reduction after treatment) was met by the colchicine group (8 vs. 2, *p*=0.017).
Diaz et al. ECLA PHRI COLCOVID^112^	1,279 patients with moderate-to-severe COVID-19: • 639 treated with SOC • 640 treated with colchicine	Colchicine: - Loading dose 1.5 mg at randomization, followed by 0.5 mg within 2 h from the initial dose, then 0.5 mg bid for 14 days or discharge, whichever occurred first. - If eGFR <50 mL/min/1.73 m^2^ or Child-Pugh B stage of hepatic failure, loading dose 0.5 mg, then after 3 days 0.5 mg every 72 h until day 14 or until discharge. Glucocorticoids used in 1,171 patients (91.5%)	The first co-primary outcome (composite of a new requirement for mechanical ventilation or death evaluated at 28 days) occurred less frequently in the colchicine arm vs. SOC (25.0% vs. 29%; HR 0.83, 95% CI 0.67–1.02, *p*=0.08). The second coprimary outcome – 28-day death – was less often observed among patients treated with colchicine (20.5% vs. 22.2%; HR 0.88, 95% CI 0.70–1.12). Diarrhea was more frequently observed in those using colchicine (11.3% vs. 4.5%).

Abbreviations

AE: adverse event. AKI: acute kidney injury. bid: bis in die. CI: confidence interval. COL-CORONA: COLchicine CORONAvirus SARS-CoV-2. COL-COVID: Colchicine in Recently Hospitalized Patients with COVID-19. COLORIT: COLchicine versus Ruxolitinib and Secukinumab in Open-label Prospective Randomized Trial in Patients with COVID-19. COV-AID: Treatment of COVID-19 Patients With Anti-interleukin Drugs. COVID-19: coronavirus disease 2019. CORIMUNO-ANA-1: Cohort Multiple Randomized Controlled Trials Open-label of Immune Modulatory Drugs and Other Treatments in COVID-19 Patients-Anakinra trial. CrCl: creatinine clearance. CrI: credible interval. CYP3A4: cytochrome P450 3A4. eGFR: estimated glomerular filtration rate. GRECCO: GReek Study in the Effects of Colchicine in Covid-19 cOmplications Prevention. HFO: high-flow oxygen. HR: hazard ratio. IMV: invasive mechanical ventilation. IV: intravenous(ly). OR: odds ratio. PCR: polymerase chain reaction. RECOVERY: Randomised Evaluation of COVID-19 Therapy. REMAP-CAP: Randomized Embedded Multifactorial Adaptive Platform for Community-acquired Pneumonia. RNA: ribonucleic acid. RR: rate ratio. SAVE-MORE: suPAR-guided Anakinra treatment for Validation of the risk and Early Management Of seveRE respiratory failure by COVID-19. SC: subcutaneous(ly). SHOCS-COVID: Symptomatic Hospital and Outpatient Clinical Scale for COVID-19. SOC: standard-of-care. SOC: standard-of-care. suPAR: soluble urokinase plasminogen activator receptor. tid: ter in die. WHO-CPS: World Health Organization Clinical Progression Scale.

Ucciferri *et al.* first showed that, in a small group of hypoxemic COVID-19 patients, canakinumab rapidly reduced CRP levels in the first 3 days, and increased the ratio of arterial oxygen partial pressure to fractional inspired oxygen (PaO_2_/FiO_2_) in the first 7 days of treatment compared to SOC alone.^91^ No adverse events, such as neutropenia or bacterial sepsis, have been recorded in canakinumab-treated patients. Other small studies reported positive experiences with canakinumab with limited adverse events^92^, ^93^, ^94^ (Table 1). To date, only two RCTs explored canakinumab in patients with COVID-19. In phase 3, multicentre, randomized, placebo-controlled CAN-COVID (Study of Efficacy and Safety of Canakinumab Treatment for CRS in Participants With COVID-19-induced Pneumonia) trial, similar proportions of patients progressed to invasive mechanical ventilation in the two arms (11.2% vs. 14.3% in canakinumab and placebo, respectively, *p*=0.29),^95^ yet treatment with canakinumab was associated with a significant reduction in the composite of death, need for mechanical ventilation or use of other immune modulators (tocilizumab/anakinra) used as rescue therapy.^95^ The three C study (canakinumab in COVID-19 cardiac injury) was a double-blind randomized proof-of-concept trial that enrolled 45 patients with moderate COVID-19, myocardial injury (defined by a troponin greater than 99% upper reference range without signs or symptoms of acute myocardial ischemia), and CRP >50 mg/L.^96^^,^^97^ Fifteen patients received a single dose of canakinumab 600 mg intravenously, 14 a single dose of canakinumab 300 mg intravenously, and 16 placebo. The primary efficacy outcome of clinical improvement (defined as the time in days from randomization to either an improvement of two points on a seven-category ordinal scale or discharge from the hospital, whichever occurs first) at day 14 was not met, nor was decreased mortality at day 28, though patients treated with high-dose canakinumab were numerically more likely to demonstrate clinical improvement at day 28 in this proof-of-concept RCT.^97^

No trials with rilonacept in COVID-19 have been reported to date.

Colchicine is an alkaloid extracted from the *Colchicum autumnale* plant and is currently used for gout, familial Mediterranean fever, and acute and recurrent pericarditis. The mechanism of action of colchicine relies on its interference with microtubule polymerization that blocks monocyte and neutrophil chemotaxis.^98^ More recently, multiple studies have shown that colchicine may exert anti-inflammatory effects through its non-specific inhibitory activity on NLRP3 inflammasome activation . Multiple observational studies exploring colchicine in COVID-19 provided overall encouraging results^99^, ^100^, ^101^, ^102^, ^103^, ^104^ and are summarized elsewhere.^98^ Scarsi et al. showed that colchicine reduced the proportion of patients who died and the risk for mortality compared with those treated with SOC alone.^99^ As well, long-term survival was also improved.^105^ In a small observational study from Italy, 9 COVID-19 outpatients received colchicine after 3–5 days of spiking fever resistant to acetaminophen or antibiotic treatment.^101^ Normalization of body temperature occurred after 3 days, with the most common adverse event being diarrhoea that did not interfere with the completion of the treatment.^101^

Few RCTs with colchicine in COVID-19 are currently available.^106^, ^107^, ^108^, ^109^, ^110^, ^111^ The RECOVERY (Randomised Evaluation of COVID-19 Therapy) arm with colchicine was stopped due to futility.^108^ The GRECCO (The Greek Study in the Effects of Colchicine in Covid-19 cOmplications Prevention) study has shown a net clinical benefit of colchicine on top of SOC compared to SOC alone in hospitalized patients with moderate-to-severe COVID-19.^106^ In particular, a reduced proportion of patients treated with colchicine experienced a clinical deterioration compared with those on SOC alone (1.8% vs. 14%, odds ratio [OR] 0.11, 95% confidence interval [CI] 0.01–0.96, *p*=0.02).^106^ Another trial enrolling hospitalized patients with moderate-to-severe COVID-19 treated with colchicine reduced the duration of oxygen need as well as hospital stay.^107^ The ColCORONA (Colchicine Coronavirus SARS-CoV2 Trial) trial was conducted among outpatients in Canada treated with colchicine or placebo. The primary endpoint (a composite of death or hospitalization due to COVID-19 in the 30 days after randomization) was not met (OR 0.79, 95% CI 0.61–1.03, *p*=0.08).^109^ However, in the pre-specified analysis including 4,159 COVID-19 outpatients with a polymerase chain reaction-confirmed diagnosis, colchicine met the primary endpoint (OR 0.75, 95% CI 0.57–0.99, *p*=0.04) and was associated with a reduced risk for hospitalization (OR 0.75, 95% CI 0.57–0.99).^109^ Finally, the ECLA PHRI COLCOVID (Effects of Colchicine on Moderate/High-risk Hospitalized COVID-19 Patients) did not show any benefit for colchicine in hospitalized patients with moderate-to-high-risk COVID-19.^112^

A phase 2, randomized, double-blind, placebo-controlled study evaluating dapansutrile (an orally administered NLRP3 inhibitor) in patients with moderate COVID-19 is currently ongoing (NCT04540120). Approximately 80 subjects are planned to be enrolled. The primary outcome investigates the proportion of subjects with clinical deterioration, defined as having any COVID-19-related hospitalization after enrolment or both (1) worsening or persistence of shortness of breath and (2) oxygen saturation less than 92% on room air or need for supplemental oxygen to achieve oxygen saturation of 92% or greater.

## Appraising the therapeutic relevance of IL-1 blockade in COVID-19: Present and future directions

Based on the findings from the abovementioned trials, some considerations are needed. The CORIMUNO-ANA-1 was stopped early due to futility as no significant difference between groups regarding the primary outcomes was found. This suggests that anakinra may not be useful in patients with mild-to-moderate COVID-19.^87^ Nonetheless, the study had a small size, was not blinded, and there was no standardization of background glucocorticoid therapy.^87^ In addition, study participants were mildly ill, not requiring supplemental oxygen, and had a relatively low inclusion cut-off for CRP (>25 mg/L). Therefore, it appears reasonable to speculate that most patients enrolled in the CORIMUNO-ANA-1 showed signs of mild-to-moderate systemic inflammation, and therefore targeting inflammation in this setting may be of limited usefulness.^78^^,^^79^ Patients in the SAVE-MORE trial with anakinra were enrolled early during hospitalization based on increased levels of suPAR.^86^ Elevated levels of suPAR might be able to detect patients with hyperinflammation and concurrent organ damage mediated by COVID-19-associated immunothrombosis.^4^^,^^86^ The positive results might be possibly explained by the early blockade of IL-1-driven hyperinflammation. Importantly, in the SAVE-MORE trial, a similar proportion of patients between groups was receiving glucocorticoids from the study beginning. Despite this, anakinra resulted in improved survival and shortened hospital stay, suggesting an independent effect. This was not the case of canakinumab in the CAN-COVID trial, which despite a positive signal, was not able to detect statistically significant improved survival. Reasons accounting for these divergent results are different. The study may have been underpowered to detect such a difference in the primary outcome as fewer patients than anticipated experienced the outcome of interest. In addition, many patients received other cytokine inhibitors, primarily tocilizumab, and this occurred more frequently in the placebo arm than in the canakinumab group. Furthermore, the main difference between anakinra and canakinumab relies on the concomitant blockade of IL-1α and IL-1β as for anakinra, while only IL-1β for canakinumab. SARS-CoV-2 induces endothelial damage resulting in increased release of IL-1α, which is not blocked by canakinumab. In addition, patients in the SAVE-MORE trial were enrolled based on suPAR levels, and not on CRP or ferritin levels. In a post-hoc analysis of the SAVE-MORE trial, CRP, neutrophil-to-lymphocyte ratio (NLR), ferritin and aspartate aminotransferase (AST) were predictors of favorable response to anakinra suggesting that they may be used in place of suPAR when not available. Indeed, CRP greater than 100 mg/L is often considered a marker of hyperinflammation. However, it is important to consider that 146 patients with low baseline CRP (below 25.3 mg/L) but increased IL-6, ferritin, and suPAR, showed clinical benefit of anakinra^86^^,^^113^ suggesting that suPAR is superior as an early marker of hyperinflammation to detect organ damage earlier than other biomarkers. However, access to suPAR testing may be limited and for this reason researchers of the SAVE-MORE study proposed the Severe COvid Prediction Estimate (SCOPE) score.^114^ This includes levels of CRP, D-dimer, IL-6, and ferritin in patients not receiving mechanical ventilation. Indeed, the SCOPE score showed to be accurate in predicting progression to severe respiratory failure or death within 14 days when compared to suPAR leves ≥6 ng/mL (area under receiver operator characteristic curve 0.81 for both) and was validated in two similar independent cohorts.^114^ A SCOPE score ≥6 was found to anticipate clinical worsening (progression to severe respiratory failure or death within 14 days of hospitalization)^114^ and could be used as an easy tool to guide therapeutic strategies in COVID-19 patients.

It is also worth considering that, while genetic determinants may predispose to SARS-CoV-2-induced hyperinflammation, these may also affect therapeutic responses to anti-cytokine treatments, including IL-1 inhibitors. Identification of early predictors (laboratory, radiologic, clinical) of response or non-response to immunomodulation may be desirable.^115^^,^^116^ In addition, the timing of intervention appears critical, since anticipated immunomodulation may favour viral spreading while delayed immunomodulation may not be effective when tissue damage has already occurred.

It is now well-established that targeting hyperinflammation in severe-to-critical COVID-19 considerably improves clinical outcomes.^3^^,^^6^ This is witnessed by the fact that distinct immunomodulatory strategies using glucocorticoids, IL-6 receptor blockers (tocilizumab, sarilumab) and the Janus kinase (JAK) inhibitor baricitinib are beneficial, and have become the mainstay of treatment for hospitalized COVID-19 patients.^117^ Only one observational study compared IL-1 and IL-6 inhibition in COVID-19 patients and found that anakinra was more effective.^118^ Among 392 patients (275 receiving interleukin inhibitors, with 62 receiving anakinra and 55 receiving an IL-6 inhibitor), IL-1 inhibition with anakinra, but not IL-6 inhibition with tocilizumab or sarilumab, significantly reduced mortality in hospitazed patients with COVID-19-related respiratory failure and hyperinflammation. Protective effects of IL-6 inhibition were only observed in patients with very high CRP levels at baseline.^118^ Therefore, it is reasonable to speculate that many clinical trials, including those testing IL-1 inhibition, failed to detect potentially beneficial effects due, at least in part, to limitations inherent to trial design and conduction rather than to a lack of effect of the proposed intervention.^119^

## Conclusion

Several lines of evidence suggest that IL-1 and the NLRP3 inflammasome may play a role in the pathophysiology of COVID-19. It has been proposed that the two IL-1 isoforms, namely IL-1α and IL-1β, are implicated at different stages. IL-1α is predominantly released by epithelial and endothelial cells acting as an alarmin, thus triggering NLRP3 inflammasome activation.^76^ Excessive inflammasome activation results in abundant IL-1β release and subsequent systemic hyperinflammation, contributing to pulmonary damage, and potentially to endotheliopathy and coagulopathy, hence resulting in widespread organ damage as observed in severe COVID-19.^12^ As different IL-1 blockers are already indicated for the treatment of various inflammatory conditions, early in the pandemic they have been repurposed for the treatment of COVID-19. Initial small observational studies exploring IL-1 blockade with anakinra, canakinumab or colchicine provided promising results that have been only partially confirmed by RCTs. More recently, in the SAVE-MORE trial, anakinra was shown to significantly reduce hospital stay and improve survival and did so in a safe fashion. Based on the positive results of this trial, the EMA has recommended the approval of anakinra for the treatment of selected high-risk adult COVID-19 patients exhibiting increased suPAR plasma levels and requiring oxygen supplementation.^19^

## Outstanding questions

In order to further improve the inhibition of the dysregulated inflammatory reaction triggered by SARS-CoV-2 infection, it would be worth considering whether a more specific inhibition of the steps preceding NLRP3 inflammasome oligomerization might be beneficial. To date, a number of experimental NLRP3 inflammasome inhibitors are currently available (MCC950, BAY 11–7082, INF4E, 16,673-34-0, OLT1177 [dapansutrile], CY-09, tranilast), as detailed elsewhere.^120^ OLT1177 (dapansutrile) is an orally active specific NLRP3 inhibitor which has been studied in gout and HF.^121^, ^122^, ^123^ A randomized controlled trial testing the safety and efficacy of dapansutrile in moderate COVID-19 (NCT04540120) is currently ongoing and results are expected. It is then conceivable that NLRP3 inflammasome inhibition might blunt the inflammatory cascade triggered by SARS-CoV-2 infection by blocking different inflammatory mediators produced by the inflammasome. Currently available data from early phase clinical trials with specific NLRP3 inflammasome inhibitors in the cardiovascular field have shown promising results awaiting validation. Results from dedicated clinical trials are eagerly needed to see whether these results would apply also to COVID-19.

## Contributors

NP, MGDB, AA, and AB conceived the general structure of the manuscript. NP, MGDB, AV, and AB drafted the first version of the manuscript. AB drafted Figure 2 and obtained permissions to reproduce other figures. RC, PCC, EP, DDG, FD, and AA provided critical revisions of the first draft of the manuscript. All authors read and approved the final version of the manuscript.

## Declaration of interests

PCC has been on scientific advisory committees for Kiniksa Pharmaceuticals Ltd., Bristol Myers Squibb and Sobi and has received grants from Kiniksa Pharmaceuticals Ltd. and Novartis.

AV received a travel grant from Kiniksa Pharmaceuticals Ltd. to attend the 2019 AHA Scientific Sessions and honoraria from Effetti s.r.l. (Milan, Italy) to collaborate on the medical website www.inflammology.org.

AA received research support from Kiniksa Pharmaceuticals Ltd., Novartis, Olatec, R-Pharm, Serpin Pharma, served as an advisor to Abiomed, Applied Clinical Intel, Cromos Pharma, Eli Lilly, Implicit Bioscience, Janssen, and Novo-Nordisk, and has received honoraria from Effetti s.r.l. (Milan, Italy) and Kiniksa Pharmaceuticals Ltd. for educational events.

AB received a travel grant from Kiniksa Pharmaceuticals Ltd. to attend the 2019 AHA Scientific Sessions and honoraria from Effetti s.r.l. (Milan, Italy) to collaborate on the medical website www.inflammology.org.

The remaining authors have nothing to disclose related to this study.

## Appendix Supplementary materials

Image, application 1

Image, application 2

Image, application 3

Image, application 4
