# Supplementary material for: Interleukin-1 and the NLRP3 inflammasome in COVID-19: Pathogenetic and therapeutic implications
Source: eBioMedicine. 2022 Oct 6;85:104299. doi: 10.1016/j.ebiom.2022.104299 (PMC9536001; doi:10.1016/j.ebiom.2022.104299)
Supplement: Supplementary file 3 [file mmc3.docx]

**Supplementary Table 1. Diseases for which therapeutic strategies with IL-1 blockade have been investigated**
