# Supplementary material for: Interleukin-1 and the NLRP3 inflammasome in COVID-19: Pathogenetic and therapeutic implications
Source: eBioMedicine. 2022 Oct 6;85:104299. doi: 10.1016/j.ebiom.2022.104299 (PMC9536001; doi:10.1016/j.ebiom.2022.104299)
Supplement: Supplementary file 4 [file mmc4.docx]

**Table 1. Diseases for which therapeutic strategies with IL-1 blockade have been investigated**

| ***Joint, bone and muscle diseases*** |
| --- |
| Rheumatoid arthritis |
| Ankylosing spondylitis |
| Psoriatic arthritis |
| Gout and pseudogout |
| Osteoarthritis (in particular in erosive osteoarthritis of  the hand) |
| ***Auto-Inflammatory diseases*** |
| Cryopyrin-associated periodic syndrome (CAPS) |
| Familial Cold Auto-inflammatory Syndrome (FCAS) |
| Muckle-Wells Syndrome (MWS) |
| Familial Mediterranean fever (FMF) |
| Deficiency of the interleukin-1-receptor antagonist (DIRA) |
| Pyogenic arthritis, pyoderma gangrenosum and acne syndrome (PAPA) |
| NLRP12 autoinflammatory syndrome (NLRP12AD) |
| Tumor necrosis factor receptor-1-associated syndrome |
| Hyperimmunoglobulinemia D and periodic fever syndrome (HIDS)/mevalonate kinase deficiency (MKD) |
| Adult-onset Still’s disease |
| Schnitzler syndrome |
| Juvenile idiopathic arthritis |
| Behçet’s disease |
| ***Cardiovascular diseases*** |
| Pericarditis (acute, recurrent and constrictive pericarditis) |
| Acute myocardial infarction |
| Acute heart failure |
| Heart failure with reduced and preserved ejection fraction |
| Secondary prevention of cardiovascular disease |
| Myocarditis |
| Stroke  Pulmonary arterial hypertension |
| ***Metabolic diseases*** |
| Diabetes mellitus |
| ***Infective and inflammatory diseases*** |
| Septic shocks complicated by ARDS, DIC, HLH or MAS |
| COVID-19 |
| ***Malignances*** |
| Breast cancer |
| Myeloma |
| Lung cancer |
| ***Neurological diseases*** |
| Amyotrophic lateral sclerosis |
| Alzheimer disease |
| Subarachnoid haemorrhage |
| Multiple sclerosis  Neuromyelitis optica |
| **Others** |
| Hidradenitis suppurativa |
| Graft versus host disease |
| Hidradenitis suppurativa |
| Steroid-resistant autoimmune inner ear disease |
| Dry eye syndrome |
| Pustular psoriasis |
| Severe atopic dermatitis |

**Abbreviations:** ARDS: acute respiratory distress syndrome; DIC: disseminated intravascular coagulation; HLH: hemophagocytic lymphohistiocytosis; MAS: macrophage activation syndrome.
